# Identifying barriers and improving communication between cancer service providers and Aboriginal patients and their families: the perspective of service providers

**DOI:** 10.1186/1472-6963-13-460

**Published:** 2013-11-04

**Authors:** Shaouli Shahid, Angela Durey, Dawn Bessarab, Samar M Aoun, Sandra C Thompson

**Affiliations:** 1 Combined Universities Centre for Rural Health (CUCRH), University of Western Australia, Perth, Western Australia; 2 Aboriginal Health Education Research Unit, Curtin Health Innovation Research Institute, Curtin University, Perth, Western Australia; 3 School of Nursing and Midwifery, Faculty of Health Sciences, Curtin University, Perth, Western Australia

**Keywords:** Aboriginal, Indigenous, Cancer, Communication, Health service provider, Cancer service provider

## Abstract

**Background:**

Aboriginal Australians experience poorer outcomes from cancer compared to the non-Aboriginal population. Some progress has been made in understanding Aboriginal Australians’ perspectives about cancer and their experiences with cancer services. However, little is known of cancer service providers’ (CSPs) thoughts and perceptions regarding Aboriginal patients and their experiences providing optimal cancer care to Aboriginal people. Communication between Aboriginal patients and non-Aboriginal health service providers has been identified as an impediment to good Aboriginal health outcomes. This paper reports on CSPs’ views about the factors impairing communication and offers practical strategies for promoting effective communication with Aboriginal patients in Western Australia (WA).

**Methods:**

A qualitative study involving in-depth interviews with 62 Aboriginal and non-Aboriginal CSPs from across WA was conducted between March 2006 - September 2007 and April-October 2011. CSPs were asked to share their experiences with Aboriginal patients and families experiencing cancer. Thematic analysis was carried out. Our analysis was primarily underpinned by the socio-ecological model, but concepts of Whiteness and privilege, and cultural security also guided our analysis.

**Results:**

CSPs’ lack of knowledge about the needs of Aboriginal people with cancer and Aboriginal patients’ limited understanding of the Western medical system were identified as the two major impediments to communication. For effective patient–provider communication, attention is needed to language, communication style, knowledge and use of medical terminology and cross-cultural differences in the concept of time. Aboriginal marginalization within mainstream society and Aboriginal people’s distrust of the health system were also key issues impacting on communication. Potential solutions to effective Aboriginal patient-provider communication included recruiting more Aboriginal staff, providing appropriate cultural training for CSPs, cancer education for Aboriginal stakeholders, continuity of care, avoiding use of medical jargon, accommodating patients’ psychosocial and logistical needs, and in-service coordination.

**Conclusion:**

Individual CSPs identified challenges in cross-cultural communication and their willingness to accommodate culture-specific needs within the wider health care system including better communication with Aboriginal patients. However, participants’ comments indicated a lack of concerted effort at the system level to address Aboriginal disadvantage in cancer outcomes.

## Background

Effective communication between patients and providers is essential to achieve positive health outcomes for users of services [1-3]. Good communication is particularly vital for successful cancer treatment as patients will have multiple anxieties at different stages of their cancer journey. How an individual experiences and copes with cancer depends not only upon the type of cancer and the treatments available, but on social support in a variety of forms, including mechanisms for dealing with uncertainty. A patient’s ability to participate in decision-making around treatment also plays a major role in satisfaction with care [4]. Most patients and families experiencing cancer go through several forms of physical, social, economic and psychological trauma. Cancer care thus encompasses long-term whole-of-care for a patient evolving much more than just managing physical and clinical symptoms going well beyond the confined hospital setting to include home and community contexts. Thus, unless information is conveyed effectively from the beginning and understood by patients and their families, it can negatively impact upon their psychological and physical health and satisfaction with care [3]. Successful communication requires co-constructing meaning to achieve agreement between the healthcare provider and patient, both of whom bring their individual physical, social and cultural orientation, prior knowledge, experiences, verbal and non-verbal communication styles [5].

Communication between Indigenous^a^ patients and non-Indigenous health service providers has been identified as an impediment to good Indigenous health outcomes [6-9]. For Indigenous Australians, the interplay of factors in the meaning-making process is more complex, occurring within the broader historical, social and political context where Indigenous experiences of marginalization, colonization, and disempowerment have led to distrust, intergenerational grief and trauma [10] resulting in a lack of acknowledgement of some of these experiences within mainstream health care. These views are consistent with the ecological model of health, and intervention to improve understanding and address Indigenous health issues will be strengthened by acknowledging that context [11].

Given the increased commitment of the Australian government to reducing the difference in life expectancies between Indigenous and non-Indigenous Australians through partnerships, gaps in understanding and difficulties in patient-provider communication warrant careful analysis. A previous study which explored Aboriginal people’s perspective on cancer care identified numerous barriers contributing to miscommunication in cancer services [12]. These included: service providers’ lack of understanding of the historical context of Aboriginal people; cross cultural misunderstanding and a lack of recognition of Aboriginal beliefs and “ways of doing things”; complex centralised health systems; logistical challenges in accessing health services [13]; linguistic differences in communication styles and a lack of trust [12]. These issues highlighted that, along with cultural competency at the clinical, organizational and systemic levels of a service delivery [14], acknowledgement of the differences between Aboriginal and non-Aboriginal culture, recognition and addressing the power differentials between the patient and service providers, and acknowledgment of the impact of history, political, social conditions on the health of Aboriginal people [15] must be considered to overcome the communication barrier between Aboriginal people and the service providers. Above all, culturally secure services that link awareness of cultural issues to actions that address them at the policy, organisational and system level [16] are needed to support Indigenous participation in cancer treatment.

Service providers need encouragement and support to reflect on and identify limitations arising in their service capability to better address and understand Indigenous needs. Lack of confidence and fear of naming deficits in their service delivery and practice can impede providers’ ability to deliver improved services that meet Indigenous people’s needs [17]. The current study was prompted as a follow-up to an earlier one [12,18,19] which explored Aboriginal Australian views about cancer and cancer services and the barriers they identified in accessing cancer services in WA. Our aim was to explore the issues of Aboriginal people accessing cancer care from the perspective of Cancer Service Providers (CSPs), since improving Aboriginal cancer outcomes requires a common understanding of both patients’ and providers’ perspectives that need to be understood and resolved for the health system to function optimally and in culturally sensitive manner for the care of Aboriginal people with cancer. This paper presents an analysis of interviews with CSPs and their experiences working with Aboriginal cancer patients and discusses issues that emerge, directly or indirectly, related to their practice and which affect Aboriginal patient-provider communication. It also presents strategies suggested by CSPs for promoting effective communication with Aboriginal patients in WA.

## Methods

This study was approved by the Human Research Ethics Committee of Curtin University, WA Country Health Services and the Western Australian Aboriginal Health Ethics Committee.

### Recruitment of participants and data collection

Data collection occurred in two phases – between March 2006 and September 2007 and April and October 2011. Initially, a formal letter containing the information sheet and consent form were sent to service managers to circulate information about the project to staff, requesting that interested staff contact the researchers. After being contacted by potential participants, the interviewer discussed the study and arranged a time for an in-depth interview. Other participants were purposively recruited from a range of locations, services and professions. Informed consent was provided by all participants.

The semi-structured interview schedule used in the previous research [12,18,19] was reviewed and modified. This covered questions around health service providers’ general experience with Aboriginal patients with cancer, any specific issue they face and possible solutions to these issues. As communication was identified as one of the major barriers by Aboriginal participants in the earlier study, we included a specific question related to communication with Aboriginal patients and their families for this study.

### Data analysis

The interviews were digitally recorded, transcribed verbatim and imported into N-Vivo software to assist management of data. Transcripts were analysed by organising and coding the data into themes which were ordered, recorded and stored as ‘free nodes’ from which other themes were developed through a process of line-by-line reading of the transcriptions [20]. The frequency and distribution of important themes were identified, highlighted, grouped and stored as ‘tree nodes’ which enabled a series of sub-themes to be developed under the key theme. Trustworthiness of the data was ensured through searching for rival explanations and linking the findings and conclusions to data, theory and evidence from the literature [21-23]. In addition, inter-coder reliability checks of interview analysis were conducted by members of the research team to check and re-check coding [22].

The design, framing and analysis of this research was based upon the philosophical foundations of the social constructionist perspectives that guided us to understand how people interpret and make sense of experiences within particular socio-cultural, political and historical contexts [11,24]. The interpretation and analysis of findings was guided also by theoretical standpoints that included the social ecological framework [25], the ‘critical cultural approach’ [26], ‘cultural security’ framework [16] and concepts of Whiteness and privilege [27,28]. These theories are all relevant to the interpretation of the challenges in communication faced by non-Aboriginal health professionals working with Aboriginal patients and their families. We were aware of the common tendency to frame findings in a way that suggests “deficiency” in Aboriginal people or communities [29] and have instead presented a range of responses identifying various levels of awareness about the role of health services and providers play in promoting or undermining Aboriginal health and wellbeing. We anticipated these approaches would identify changes needed to current practices to promote good communication with Aboriginal people.

## Results

CSPs having experience working with Aboriginal patients were recruited from a variety of service settings: hospitals, the Cancer Council WA, cancer screening services, palliative care services, Aboriginal community health services and other relevant allied health services providing support to cancer patients in Perth and six rural/remote regions of WA. Sixty-two in-depth interviews were conducted of whom 17 participants were Aboriginal CSPs; participants’ demographic characteristics are described in Table 1.

**Table 1:** Demographic information about the CSPs

**Positions or roles**	
Clinical (Nurses and GPs)	18
Oncologist/Radiation Oncologist	2
Aboriginal Liaison Officers (ALOs)/Aboriginal Health Workers (AHWs)	10
Cancer Nurse Coordinators	5
Social Workers	7
Aboriginal Community Health Nurse	2
Palliative Care Providers (PCPs)	11
Others	7
Total	62
**Place**	
Urban	33
Rural	29
Total	62
**Gender**	
Female	51
Male	11
Total	62
**Aboriginality**	
Aboriginal	17
Non-Aboriginal	45
Total	62

Key themes that emerged as barriers to communication for CSPs were their lack of knowledge about the cultural, social and health needs of Aboriginal people; the marginalization of Aboriginal people within the system; and Aboriginal patients’ distrust of the health system and fundamental aspects of communication, including language and communication style.

Strategies identified by both Aboriginal and non-Aboriginal CSPs to improve CSPs’ communication with Aboriginal patients included: recruitment of more Aboriginal staff; cultural training for the CSPs; education for Aboriginal stakeholders; providing assistance to the patients during patient consultations with clinicians, with careful and repeated explanations; continuity of care; clear, simple and empathic communication; and coordination within services. These will be discussed, sub-themes described and the similarities and differences in responses with the previously reported Aboriginal patients and family participants [12] identified (Table 2).

**Table 2:** Differences and similarities in Aboriginal and non-Aboriginal participants’ views on barriers to communication

**Barriers to communication**	**Cancer service providers**		*Aboriginal patients/families
	**Aboriginal**	**Non-Aboriginal**	
**Contextual barriers**			
•→Racism and colonisation	X		X
•→Aboriginal marginalization	X	X	X
•→Paternalistic attitude of the CSPs	X	X	X
•→Lack of understanding by CSPs about Aboriginal needs		X	
•→Lack of coordination within the services		X	
•→Lack of Aboriginal staff	X	X	X
**Impediments to communication**			
•→Language	X	X	X
•→Communication style		X	X
•→Privacy and confidentiality	X	X	X
•→Concept of time		X	
**Loss of trust**			
•→Distrust	X		X

*Aboriginal patients and family affected by cancer [12].

### Contextual barriers to communication

#### Lack of understanding by CSPs about the overall needs of Aboriginal people

One challenge reported by non-Aboriginal providers was their lack of knowledge and understanding of the complex multiple issues that Aboriginal people faced in their day-to-day lives, including unsuitable housing for patients when discharged. These often led to misunderstanding patients’ needs during consultations.

*I suppose this latest young lady, well one of the frustrations I had was organising to get her to the metro area and highlighting all of the possible complexities with her situation being a young woman with young children and an extremely nasty cancer but not getting the support from some of the hospital staff in the metro and then not, I guess they basically turned away, some of the supports that I had linked the patient with. As when those people went to get in touch and be involved they said, “Your services aren’t needed.” And because of that, and I don’t know … they certainly had no understanding of the complexities of what she would face when she came back home.* (CSP13, Rural non-Aboriginal nurse)

Several participants reported making assumptions about Aboriginal cultural preferences such as preferred place of stay at the end-of-life, gender-specific communication, and the appropriateness of talking about cancer or death and dying that led to increased concern and vigilance: “*we do not communicate with them, we will be excessively cautious and respectful…. There’s a lot of uncertainty and a lot of confusion*” (CSP54, urban non-Aboriginal participant). In response to questions about why they did not ask for clarification, CSPs often indicated they thought that would be inappropriate. Thus, many of the practical issues that Aboriginal patients faced were not explored or were not addressed in an appropriate manner.

Another challenge for services was consulting and negotiating with extended family members to obtain consensus about treatment and end-of-life decision-making. This sometimes impeded timely delivery of healthcare to the patient as service providers often needed consent from multiple people, some of whom often were present but at certain times extended family members were located in remote communities.

#### Lack of understanding of the Western medical system by Aboriginal patients

Both Aboriginal and non-Aboriginal CSPs perceived Aboriginal people’s lack of understanding about cancer and the medical system as a major impediment to communication. All participants reported that most Aboriginal patients and families struggled with understanding how the Western medical system worked. CSPs found that, particularly rural and remote Aboriginal patients and their families lacked understanding of the importance of adhering to oncology treatment regimes, the need to attend follow up appointments on time; the length of stay for treatment; the importance of staying near the place of treatment and abstaining from smoking and consuming alcohol. These resulted in misunderstanding and frustration among non-Aboriginal CSPs and silence in Aboriginal patients, which ultimately contributed to further breakdown in communication.

*That is a bit of struggle, the communication is really difficult because Aboriginal people don’t have a voice, they’re reluctant to use their voice and even with my support, they still find it difficult to actually [communicate]* (CSP28, rural Aboriginal participant)

Mostly non-Aboriginal participants reported difficulty in explaining the importance of treatment and follow-up care to Aboriginal patients:

*Him understanding what I was wanting to do with him and because he had insulin at certain times of the day and he’d gotten into that routine he thought it was my duty of care to say to him, “Well actually, we do it in hospital like this, and we have to give you your treatment and we have to give you your medications”, and … he didn’t understand what I was saying to him, that’s why he was getting angry and anxious. It’s understandable.* (CSP9, Urban non-Aboriginal participant)

While the problem was often externalized onto the Aboriginal patient, the often inappropriate communication of health providers was identified by Aboriginal participants:

*They [non-Aboriginal health professionals] need to come down a peg. Instead of talking high and mighty to a person … they should sit down and relax and actually talk English to that person instead of using big words, fancy words, making themselves look good and making themselves feel proud that they can, you know, ‘I know what this word is, but you don’t because you are small.’* (CSP49, Rural Aboriginal participant)

Some CSPs acknowledged the diversity among Aboriginal people based on their educational background, area of residency and their degree of acculturation. The analysis revealed that Aboriginal and non-Aboriginal CSPs with longstanding experience and relationships with Aboriginal communities understood patients’ issues sooner and had confidence in addressing them empathetically and in a manner considered to be more culturally appropriate: “*without being kind of aggressive about it, you have to dig a bit to really find out about the person… health needs and problems”* [CSP26, urban non-Aboriginal participant]. Due to the pressures on clinicians working within health, they often did not want to unearth patient’s contextual issues, recognising that they would need more time to address them:

*… if you’re busy you know, and you don’t want to ask those questions because if the answer comes back positive, then what do you do? I certainly have come across people who say they don’t want to ask that question because then you’ll… you can be another half hour there, yes. But yes, I think there’s a bit of what are you there for really?* (CSP26, urban non-Aboriginal participant)

#### Marginalisation of Aboriginal people and distrust

Aboriginal CSPs mentioned that history, racism and associated distrust towards the medical system by Aboriginal people are exacerbated by poor communication with non-Aboriginal CSPs. Service providers’ control of the environment, structure, purpose, topic and language of communication highlighted their position of power, authority and reinforced the marginalised position of Aboriginal patients within the system:

*… lack of insight I think from the nurses there and inappropriate sense of possession or ownership or I don’t know, superiority in their own skills and abilities which is unfounded. Their lack of accepting that people might need more than just them to help, to deal with their situation. They’re very insular and they feel that they are a one stop shop and they can provide everything but they can’t.* (CSP13, rural non-Aboriginal participant)

Interestingly, this critique of the inability of some CSPs to recognise and respond to the power imbalance, fits well with whiteness theory, where whiteness is normalised, taken for granted and therefore acts as an invisible form of privilege and superiority. Some non-Aboriginal providers reported counterbalancing this distrust by actively building relationships and investing time in empathic listening to Aboriginal patients. However, several were unsure about the process and reported that building trust often took a long time. This highlights the needs for the system level change to support the service providers so that they can work towards the goal of achieving culturally safe services for Aboriginal people. Non-Aboriginal providers reported the feeling that they were likely to fail when dealing with Aboriginal patients, because Aboriginal people did not fit the usual models and for their care there was a clash with the demands of the system in which they worked: “*when you know you are gonna have an Aboriginal person coming to your clinic, your heart sinks….”* (CSP26, urban non-Aboriginal participant) This participant, as with some other non-Aboriginal CSPs, felt they lack the power to improve Aboriginal people’s participation in cancer treatment, but there was relatively little interrogation from their point of view of how the system could be changed to accommodate the Aboriginal patient’s needs or even the power to make a difference to an individual patient through the quality of the relationship that they developed with an Aboriginal patient and their family.

### Impediments to communication

Fundamental issues related to communication which were highlighted were language, communication styles and differences in the concept of time.

#### Language

Language was a barrier that led to misunderstandings between Aboriginal patients and clinicians as some Aboriginal patients communicated in Aboriginal English which often differs from standard Australian English in pronunciation and meaning. Other misunderstandings were the result of medical professionals’ difficulty in communicating with Aboriginal patients in clear, jargon-free language so they are understood. Aboriginal and non-Aboriginal CSPs criticised the poor patient interaction skills of some medical professionals which added to the distrust experienced by Aboriginal patients:

*Oh he’s just an ignorant, bit of a dickhead. … [Non-Aboriginal patients] might admire his competence, but they don’t like his manner… we have a couple of oncologists like that and they’re very good oncologists but they’re … (laughter)… hideous communicators. Non-Aboriginal people will just think, “Oh” but they’ll accept that because of his competence whereas Aboriginal people just won’t go back.* (CSP27, rural non-Aboriginal participant)

This comment illustrates how for those who are comfortable within the Western model of care, the technical competence of a clinician may be enough for them to disregard what to an Aboriginal patient may be considered as further evidence of a system in which they are uncomfortable and therefore not likely to feel well cared for, particularly in a holistic sense.

#### Communication style

Most non-Aboriginal CSPs described Aboriginal people as ‘non-assertive’, ‘non-responsive’, shy to ask questions and reluctant to talk about their illnesses, accompanying problems and reactions to what was happening in their lives. According to some, Aboriginal people lacked enough education and confidence to ask appropriate questions, evidenced by the tendency of patients to agree with whatever was being said (referred to in the literature as ‘gratuitous concurrence’) [7]: *“it was difficult because often people would say ‘yes I understand’ and you don’t know if they did”* (CSP3, urban non-Aboriginal participant). There was little consideration by CSPs that Aboriginal acquiescence could be a learnt response as a result of years of marginalization and oppression and having being subdued in a way that has silenced their voices [11], a response that would be predicted by the socio-ecological model [25]. Aboriginal people were labeled as ‘non-responsive’ because the Western medical system generally expects individuals to conform to the system, assert their preferences and to take control of their personal health care.

Interestingly, when Aboriginal providers communicated with non-Aboriginal patients and families they found them reserved, withholding their thoughts with them. The willingness to share information was noted as different when dealing with an Aboriginal provider. One Aboriginal CSP explained how she felt ‘known’ and close when she dealt with an Aboriginal person. Aboriginal staff within the services often provided a “zone of comfort” for Aboriginal patients “*… there was a patient here who wouldn’t even see the doctor unless I was in the room and with them”* (CSP1, urban Aboriginal participant). Optimal caregiving and advice seemed to occur when patients and providers shared the same cultural background, and life experiences, highlighting the importance of cultural security for improving outcomes.

#### Differences in the concept of time

Mainstream service providers struggled to adjust to differences in the ‘concept of time’ used by Aboriginal people which hampered communication both directly and indirectly. Moreover, CSPs who work part time mostly find it difficult to meet the needs of Aboriginal patients who, often, do not operate within a time schedule where appointments are diarized:

*With that particular case we’d already set up a meeting with the Aboriginal Health Service and then the workers of the Health Service had made other appointments and gone off. So we all finally got there, but the actual Aboriginal Health Service wasn’t free and then that blows your mind. You think, “Oh my God.” …so in many ways it takes an awful lot longer. You’ve really got to build more bridges and go more slowly and be more fluid in how you work which for me is difficult because I only have two days in which I can do anything with anybody so I can’t be as fluid.* (CSP56, Rural non-Aboriginal participant)

CSPs operated by allocating a specific time for each patient in advance, and *“that doesn’t always meet people’s needs”* (CSP14, rural non-Aboriginal participant). One participant said, *“When you’re dealing with (particularly more traditional) Aboriginal patients … they don’t live by a clock. They don’t sort of come to appointments on time…. So I think the difficulty of providing good care as an outpatient is partly based on whether we can find the patients in a timely fashion on the day that I’m visiting”* (CSP52, urban non-Aboriginal participant). This elastic time particularly created difficulties with video conference consultations. Some CSPs tried to find a solution and they booked their appointments “*for half an hour either side for the consult, so that we can ensure that the patient is actually seen timely*” (CSP14, rural non-Aboriginal participant)

*So that’s a huge difficulty but it’s not with all Aboriginal families. Some families you can say I’ll be there at two, and everybody is there and they’re quite happy to sit down and meet, it’s just particular families can be quite difficult.* (CSP56, Rural non-Aboriginal participant)

### Strategies to improve communication

#### Aboriginal staff and support people within services

CSPs expressed the need for more Aboriginal staff who, in their view, enhanced the quality of communication with Aboriginal patients: “*at the moment I find them [Aboriginal patients] not easy opening up to us and talking to us about any psycho-social problems [but] they feel more comfortable talking to some of their own people… might be their relatives at home*” (CSP50, rural non-Aboriginal participant). An informed and responsible family member escorting the patient or an Aboriginal staff member could help non-Aboriginal providers better understand patients’ needs and circumstances. It also made easier for Aboriginal people to trust the health system and highlighted benefits of establishing and developing partnerships between health services and the Aboriginal community. The role of Aboriginal service providers as cultural brokers was particularly significant when CSPs dealt with patients in outreach areas as Aboriginal staff often knew the “ins and outs” of the community, such as where certain people lived and what was happening with them at that time. Their informed cultural and local knowledge was extremely valuable and provided an ongoing resource for the CSPs.

Although many providers were aware that yarning with Aboriginal patients and families informally could help them feel more comfortable and relaxed, in most circumstances only Aboriginal staff could confidently achieve this. Interacting with Aboriginal patients and bonding with them using a conversational approach is referred to as ‘social yarning’ [30]:

*… generally with Aboriginal people it works very well to actually be able to go away from the clinical environment and go and sit under the tree and just do a bit of yarning and get to know and build a relationship.… things like home visits are really valuable* (CSP14, Rural non-Aboriginal participant)

#### Training and education

According to most participants, educating Aboriginal people about cancer and the Western medical system was important. However, increasing CSPs’ knowledge of Aboriginal culture and life-style was also considered ‘key’: “… *it comes back to knowledge again. If we all have a bit more knowledge on how we might do it better, how we might do it differently. I think we’d all be a lot better off*” (CSP56, Rural non-Aboriginal participant). Participants suggested that further emphasising cultural education within health curricula, on-going ‘on-the-job’ health-related cultural awareness training for CSPs and more interaction with Aboriginal people would increase the knowledge of CSPs. Many participants considered the available cultural awareness training insufficient as it focused too much on what had happened in the past. Access to extensive health-specific cultural training opportunities was an identified need.

#### Assistance while consulting clinicians

Communication could also be improved by Cancer Nurse Coordinators (CNCs) (positions established in 2007, prior to the second period of data collection) or an Aboriginal Liaison Officer (ALO) supporting patients during their appointments with clinicians. This support assisted patients to better understand their treatment, side-effects and symptoms. Participants reported that although it required preparation, planning, consultation and coordination between different services, the process ultimately assisted providers to be informed about the patient’s actual condition.

*We tend to offer that service where we go in with patients and we sit with them when the doctor is explaining to them and for some patients we’ll even take notes and give them to the patient afterwards in plain simple language.* (CSP6, rural non-Aboriginal participant)

#### Repeat explanations

Repeat explanations by an ALO or a CNC about a treatment and its side-effects, the importance of continuity of treatment, and consulting the patient before and after seeing their treating clinician were other strategies which helped improve patient/clinician communication. These were regarded as facilitating better adherence of patients to their treatment regime and better outcomes and were particularly useful for patients undergoing complex treatment regimes, such as for patients with lung, cervix, head and neck cancers.

*…predominantly cervical cancer, they need to have radiation therapy daily for a good six weeks as part of their treatment plus some chemo and that’s quite hard work to ensure that they complete that because it’s every day you have to be in for a fifteen minute treatment and so there’s a lot of meeting at the appointments… two or three times a week sometimes, or at least ringing them if they’ve got a mobile phone and reminding them don’t forget you’ve got your treatment, I’ll meet you today or being at the treatment. Because they don’t always turn up for one or two days. It’s sort of understanding that you can’t break your treatment*. (CSP2, Urban non-Aboriginal participant)

Clear verbal explanation, visual materials, suitable pictures and face-to-face conversations with Aboriginal patients were also identified as important: ‘*I find a picture, and I go on the internet, I find a picture, a basic picture and I bring it back and I go over the picture with them.’* (CSP1, Urban Aboriginal participant)

#### Continuity of care

Continuing care by the same CSP was recognized as important to establishing a trustworthy relationship. Aboriginal people were more comfortable when they met the same person during their visit to a service:

I: So is it important to have the same person in every visit?

*P: If it’s possible, but it’s not always possible. But also, if it’s going to happen that you don’t have the person, explain it to the patient that next week I’m not here but she [another staff member] will fill my boots and she will do exactly the same as I do, and then when I come back we can continue or this person is going to take over your care. You have to explain to them that you might move on and somebody else is going to take over then they know what’s going on. I think if it comes from you that they trust now and they see this person is good and they can manage with everything that should be just as all good for them. You can’t have a continuity of care if you want it, it’s just not possible. But you can at least try.* (CSP50, Rural non-Aboriginal participant)

#### Understanding and accommodating patients’ needs

Some participants reported that Aboriginal patients and families valued attention, support and understanding from providers, and this helped them feel relaxed and connected within the system. Accommodating the patient’s needs required CSPs being aware of the patient’s context, circumstances, background and needs. Allied health professionals played a significant role in that aspect. One non-Aboriginal CSP talked about how he built an empathic relationship gradually with one male Aboriginal patient who was suffering from head and neck cancer, had severe dental problems and a negative view of the health system. From the beginning, hospital staff tried to ensure everything fell into place to support his treatment, appointments, pain management and needs. They interacted with his extended family members, and eventually the patient and his family became comfortable, and started interacting with staff. This example showed that a positive change in a CSP’s attitudes and practice helped to build trust and improved the patient’s experience of health care, their engagement with the service and contributed to comfort during the illness.

#### Coordination within services

Non-Aboriginal CSPs reported that oncology patients generally are offered too many services, which can be overwhelming for many patients. Several participants felt that although there were many issues still to be addressed, improvements had begun with care coordination now shared among and between different services. For example, regional CNCs can communicate with patients in smaller communities via local networks and help best manage their care with appropriate referrals. With CNC support, specialized health professionals in metro areas can make informed decisions about patients living in isolated areas. CNCs in rural and remote areas can also make sure that a “*patient fully understands the appointment dates and the length of time they would be staying in the metro*” (CSP3, urban non-Aboriginal participant) before they come for treatment:

*I think with careful and good co-ordination and good networking, using the people that meet the patient’s needs… I think the whole team has learnt from that. But it is about making sure that there’s pathways and processes in place and so, yes, we’re working on that and always the issues are around the practical good communication, that’s the key factor I think.* (CSP14, rural non-Aboriginal participant)

#### Privacy and confidentiality

Arrangements to ensure privacy were mentioned by some service providers to ensure trustworthy and respectful communication between Aboriginal patients and providers, while Aboriginal patients and the family members participated in the previous study [12] identified them as crucial aspects of culturally appropriate care: ‘*it must be a quiet area, you mustn’t have lots of people coming in and out and lots of disturbances and stuff and sometimes a one-on-one with a parent or aunty there, is much better than having a big group of people… also because it just freaks them out if there’re lots of people standing around them and they don’t know what’s going to happen, especially if it goes ahead with procedures.’* (CSP52, urban non-Aboriginal participant).

## Discussion

Communication with health providers was a key barrier for Aboriginal people with cancer identified in a previous study exploring the experiences of Aboriginal people with cancer [12]. This study explored the views of CSPs (both Aboriginal and non-Aboriginal) on communication and compares the findings with those of Aboriginal patients with a view to identifying possible solutions which are sensitive to the needs of Aboriginal people as well as feasible and achievable by the service providers within their services.

The ‘cultural security’ framework ‘whereby organisations and individuals… make a positive shift away from altering attitudes and values, and move towards changing organisational and individual behaviours and practices’ [16] was one of the frameworks that guided us to assess if the prevailing Western model of care fostered good communication with Aboriginal patients and their families, responded to their needs and promoted their wellbeing. As previously indicated, cultural security recognises that theoretical ‘awareness’ of culturally appropriate service provision is not enough, and focuses directly on practice, skills and efficacy by incorporating cultural values into the design, delivery and evaluation of services. This model argues for society and system levels involvement in securing an environment that is safe for Aboriginal people, and proposes to ‘effect change in all elements of the health system workforce development, workforce reform, purchasing of health services, monitoring and accountability and public engagement’ [31]. Previous research findings drew attention to the tensions in the system where the model of standardized care that privileges Western biomedical knowledge and practice at times failed to meet the needs of Aboriginal patients, demonstrated in poor communication styles. This experience eroded an Aboriginal patient’s trust and reinforced their marginalisation. This tension was found to have intersected with the need for health providers to be flexible, respectful and responsive to cultural differences raising the issue of how such challenges were negotiated [12,13].

Findings from this research clearly indicated that some CSPs wanted to deliver high quality culturally appropriate care to Aboriginal patients yet felt powerless or did not get enough support to initiate change. They wanted to respect Aboriginal traditional beliefs and practices and incorporated some sensitivity to Aboriginal culture into their service. However, they lacked confidence and were unsure if Aboriginal patients’ needs were met. They wanted to form a relationship with Aboriginal patients but gaps remained in their understanding that compromised good communication. They reported trying to deliver health information empathically, repeating it as needed and were often prepared to spend extra time with Aboriginal patients. However, they already felt over-stretched. Where possible, extended family members were included in decision-making around treatment, however, this was challenging particularly when planning discharge and with limited resources available in the patient’s home environment to provide high quality care. Many of these positive initiatives occurred as a result of the efforts by individuals and not by the system. Therefore, while some intentions to improve Aboriginal health care were clearly stated, limitations in the system and the competing demands placed on CSPs were evident. Thus, support at the system level to ensure cultural security within health services was generally seen as missing.

Cross-cultural communication difficulties exacerbated the problem. Participants were aware of the fact that long-term relationships and trust were vital for all interactions between Aboriginal patients and their providers. However, that link was often not developed due to poor understanding of how a trusting relationship could be established or how an overloaded health system focused on throughput challenged meeting patients’ needs given busy schedules. Critical reflection by non-Aboriginal CSPs of the need for service provision to be more flexible and attuned to the needs of patients from other cultures was largely missing. While oncology patients in general have been found not to disclose concerns about their underlying beliefs which do not fit with the dominant biomedical paradigm [3], some CSPs stereotyped Aboriginal patients and associated their non-assertiveness with cultural differences, lack of education and understanding. Most of them failed to reflect back on their own short-comings in the cross-cultural communication process. Working within a health structure that privileges a Western biomedical model of care and discounts other cultural understandings of illness was not considered a factor in informing communication difficulties [32]. Often providers seemed blind to the way that delivery of cancer care is inherently racialised. Although the term “whiteness” lends itself to being associated with skin colour, Kowal [33] has pointed out that whiteness and privilege can also encompass a racialised social structure.

Overall CSPs held the view that those who understood and could work with the system were likely to have a better outcome and prognosis, referring to some Aboriginal patients as ‘coping well’, ‘articulate’ and ‘easy to deal with’. These responses reflected the implicit and pervasive expectation that Aboriginal people should adapt to the needs of the health system rather than the system becoming more flexible in meeting the needs of Aboriginal patients. Health providers’ projecting problems onto Aboriginal people and not reflecting on the limitations of their own practice highlight the need to interrogate their understanding of ‘culture’; their understanding of the socio-cultural determinants of health and the privilege positioning in health care. Moreton-Robinson [27] points out that white Anglo-Australian cultural and racial dominance is the ‘invisible omnipresent norm’ [27]: xix) in Australia and not questioned; instead it is the benchmark by which differences from that norm are measured, valued and often ignored. In a health context, this means that service providers trained in the dominant Western biomedical model of health adopt a standardised approach to care that is accepted as ‘the’ structure where Indigenous knowledge, beliefs and values are often dismissed or ignored at the level of policy and practice. This often resulted in a failure in organizational support for CSPs with inadequate education and training to understand the lived experience of many Aboriginal Australians from socio-cultural and historical perspectives.

A commitment to reflect on factors informing cross-cultural practice was needed to become aware of the impact of relations of power and authority on the marginalized position of Aboriginal patients and explore ways to improve communication so it promoted Aboriginal health and wellbeing. While the role of the AHWs is integral to providing culturally safe care to Aboriginal patients, improving the knowledge and skills of non-Aboriginal cancer service providers through education and training is equally crucial to ensure sustained improvements to practice shared by all. Historically the ‘cultural awareness’ training is being provided to improve Indigenous-mainstream relations and communication and to bridge the ‘problematic’ gaps between two distinct cultures. However, critical cultural theorists point out most of these trainings have been based on the ‘classic narrow’ view of culture that failed to see the complexity of human experiences within the broader socio-political context and can lead to a false perception of essentialising Aboriginal culture as “a unified entity called ‘Indigenous culture’ that can be described, taught and understood” [34]. As opposed to this narrow view of culture, they urge an appreciation and recognition and appreciation of culture as a relational entity which changes over time. One way forward is for Aboriginal and non-Aboriginal CSPs and key community members to work together to ensure that differing cultural values are respected, communication is improved and solutions reached in the spirit of consensus. Change requires CSPs and health services to reflect on their own cultural orientation, and identity, power and authority associated with whiteness reflected in attitudes and practices that can either promote or superior positions in the society, whiteness, identity, attitudes and practices and how these either promote or undermine Aboriginal health and wellbeing.

Respect and consideration for people from different cultures who are not fluent in English is of paramount importance in any health care, but arguably particularly for cancer care which is protracted, distressing and has multiple side effects. A raised voice and/or manifestation of anger may make Aboriginal people coming from rural and remote areas feel terrified, confused and irritated [7]. It is important for CSPs to understand that there needs to be differences in how service providers approach patients about their illness based on how patients perceive their illness. While non-Aboriginal service providers, in most circumstances, preferred short conversations focusing on issues around treatment, follow-up, medications and their disease, Aboriginal service providers used discussion, metaphors, storytelling, and pictures to connect and to build relationship and trust with their patients. This process has been identified as effective in other contexts as well [35].

In general, service providers who reported fewer communication problems with Aboriginal patients made efforts to be understanding, compassionate, accommodating, flexible and considerate. This accorded with the views of Aboriginal patients and families who expected their physicians to be “understanding”, “compassionate”, “concerned” and “empathetic” [12]. CSPs from rural/ remote regions of WA or those who had long-term experiences in dealing with Aboriginal patients were more likely to fall into this category. However, factors underpinning differences in service provider’s perspectives and approaches and how they can be affected during training or subsequently is an area requiring further research.

Underrepresentation of Aboriginal doctors and other health professionals’ further aggravates the issue. Optimal dialogue occurred when staff and clients shared the same linguistic and cultural background; when they didn’t, extra effort was required [7], and support for this was largely missing at the system level. Better communication and service delivery requires a coordinated approach and more Aboriginal staff working in the system and fully embraced by the treating healthcare team [36]. Careful attention is needed so that their roles are well defined and supported, not tokenistic. A recent study on hospitals considered to be successfully addressing the issues of their Aboriginal patients highlighted that commitment at the management level and a change at the organisational and system level is critical to success. An open, supportive and safe environment not only assisted Aboriginal staff and patients to feel comfortable within an organisation, but it also encouraged other ‘staff working to change themselves and their institutions to break down barriers’ [36]. Health services need to facilitate the process of building genuine relationships between Aboriginal and non-Aboriginal people which is the most important prerequisite for working with Aboriginal people [37].

From the standpoint of Aboriginal participants, the legacy of colonization underpins ongoing communication difficulties because of a lack of control over their lives and lack of trust [12]. Conversely, very few non-Aboriginal participants recognised that ongoing distrust could stem from ‘history and colonization’ after so many years, and would currently inhibit patient-provider communication. This shows the lack of capacity of the service providers to relate health with the factors at the macro level, such as, socio-political context, historical experiences and so on.

Often, problems with communication are attributed to Aboriginal people speaking a native tongue and having poor command of English. This is an issue for some Aboriginal people from more remote areas of the state, but the majority of Aboriginal people in WA speak English. However, it is poorly appreciated that they often use “Aboriginal English” which “differs from many other dialects of English in systematic ways at all levels of language including underlying conceptual systems and is associated with different patterns of interaction” [38], p.10. Access to professional interpreters has been identified as one approach to solving the problems in communication [7,35]; however, the diversity of Aboriginal language groups in WA creates challenges in having a professional interpreting service for each Aboriginal language. Use of interpreters or cultural brokers for different regions and groups remains largely unexplored. An accompanying family member or the availability of AHWs may be an alternative option although several problems have been identified with these approaches in the literature [7]. Suggested strategies and recommendations (see Suggestions and recommendations based on the study findings) for improving communication included fostering service provider training highlighting the social determinants of Aboriginal health and wellbeing.

### Suggestions and recommendations based on the study findings

Addressing contextual barriers

•Ensure cultural competency at the clinical, organisational and system level by

–recruiting, involving and providing training to Aboriginal staff;

–providing cultural safety training to all staff, mindful of not ‘essentialising’ culture but recognising it as fluid, dynamic, and intricately connected to a person’s social context;

–improving patient record system so that Aboriginal identification can be improved;

Make the service environment welcoming to Aboriginal people by

–consultation with the local Aboriginal communities on how their needs can be addressed within the service setting;

–displaying images that recognise and are inclusive of Aboriginal people (artwork, photography);

–having Aboriginal staff visibility including in reception areas;

–Using images and art to help explain what cancer is and cancer treatments including Aboriginal-specific resources in the display rack;

•Appropriate cancer education for Aboriginal people and evaluation of outcome

Improve continuity of care

–allow adequate time to build rapport and to get to know the person

–maximising continuity of care during treatment and follow-ups

•Provide care and follow-up closer to home where possible

•Better coordination and integration of care between cancer treatment services, primary and community-based care

Addressing specific communication issues

•Use interpreters or relevant support people to increase understanding of a patient’s concerns and needs during consultation

•Show empathy, kindness and understanding for the person and the family affected by cancer

•Use clear, plain language to communicate with Aboriginal patients and their families

•Repeat explanations around options and listen to a patient’s choices

•Make sure that Aboriginal patients and their families have understood what has been prescribed or recommended in regard to cancer treatment, follow-ups and management

Make sure that Aboriginal patients and their families have understood what has been prescribed or recommended in regard to cancer treatment, follow-ups and management

## Conclusion

Most CSPs participated in this study wanted Aboriginal patients and their families to trust them and to understand that their job is to help patients. However, a reflection of the service providers towards their own perception, positioning and the privileges they experience and a cultural shift and/or must-change within the wider health care system is required to achieve this. It is essential that non-Aboriginal providers understand that there are inherent differences in the way the English language is spoken and heard by many Aboriginal people that contributes to misunderstanding. While the Western model of health care provides service delivery to Aboriginal patients, critically reviewing its limitations is important so that service providers do not perpetuate practices that compromise the quality of care delivered to Aboriginal patients. Equally important is to develop strategies for improvement. CSPs who become aware of how their attitudes and practices may adversely impact on Aboriginal patients, and who reflect on the shortfalls in their service provision can become agents of change for service improvement at a broader systems level. As McDermott suggests ‘Becoming a thinking, culturally safe practitioner is also the prerequisite for emerging as a clinically safe one’ [39].

For services to be culturally secure, time must be spent to ensure that the patients’ needs are met, that family members are included and communicated appropriately, and that care is organised in a way consistent with their circumstances and cultural needs. This requires more than simply deciding on the most effective treatment as shown in clinical trials. When a patient feels confident that the health care worker *really cares* about them and their family and is striving to understand and respond holistically to their circumstances, the therapeutic relationship can be transformed to one which focuses on healing, not just treatment, and in which the service provider meets the needs of the patient.

## Endnote

^a^In this paper, the term Aboriginal has been used to refer to the Indigenous people of WA. We have used Indigenous when we are referring to features that are identified across different Indigenous peoples.

## Competing interests

The author(s) declare there are no competing interests, including financial and non-financial.

## Authors’ contribution

SS coordinated the project, participated in the project’s design, carried out the data collection and analysis for this project, and prepared the initial draft. AD was involved in the writing. DB, SA and SCT participated in the design, assisted with the conduct of the study, helped interpret findings, commented upon drafts of the manuscript and writing. All authors read and approved the final manuscript.

## Pre-publication history

The pre-publication history for this paper can be accessed here:

http://www.biomedcentral.com/1472-6963/13/460/prepub
